# Development and characterization of a pupal-colour based genetic sexing strain of *Anastrepha fraterculus sp. 1* (Diptera: Tephritidae)

**DOI:** 10.1186/s12863-020-00932-5

**Published:** 2020-12-18

**Authors:** José S. Meza, Kostas Bourtzis, Antigone Zacharopoulou, Angeliki Gariou-Papalexiou, Carlos Cáceres

**Affiliations:** 1 Programa Moscafrut, AGRICULTURA/SENASICA-IICA, Metapa de Domínguez, Chiapas, Mexico; 2 Insect Pest Control Laboratory, Joint FAO/IAEA Division of Nuclear Techniques in Food and Agriculture, Seibersdorf, Vienna Austria; 3 Deparment of Biology, Division of Genetics, Cell and Development Biology, University of Patras, Patras, Greece

**Keywords:** Mass rearing, Sterile insect technique, Mutation, Translocation

## Abstract

**Background:**

Area-wide integrated pest management programs (AW-IPM) incorporating sterile insect technique (SIT) have been successful in suppressing populations of different fruit fly species during the last six decades. In addition, the development of genetic sexing strains (GSS) for different fruit fly species has allowed for sterile male-only releases and has significantly improved the efficacy and cost effectiveness of the SIT applications. The South American Fruit Fly *Anastrepha fraterculus* (Diptera: Tephritidae) is a major agricultural pest attacking several fruit commodities. This impedes international trade and has a significant negative impact on the local economies. Given the importance of sterile male-only releases, the development of a GSS for *A. fraterculus* would facilitate the implementation of an efficient and cost-effective SIT operational program against this insect pest species.

**Results:**

For potential use in a GSS, three new morphological markers (mutants) were isolated in a laboratory strain of *A. fraterculus sp. 1*, including the *black pupae* (*bp*) gene located on chromosome VI. The black pupa phenotype was used as a selectable marker to develop genetic sexing strains by linking the wild type allele (*bp*^*+*^) to the Y-chromosome -via irradiation to induce a reciprocal Y-autosome translocation. Four GSS were established and one of them, namely GSS-89, showed the best genetic stability and the highest fertility. This strain was selected for further characterization and cytogenetic analysis.

**Conclusions:**

We herein report the development of the first genetic sexing strain of a major agricultural pest, *A. fraterculus sp. 1*, using as a selectable marker the *black pupae* genetic locus.

## Background

The sterile insect technique (SIT) is a species-specific and environmentally friendly genetic method to control populations of major insect pests. This method involves the rearing of the target pest species, the induction of lethal mutations and atrophy of reproductive organs to induce reproductive sterilization through the exposure to ionizing radiation, in the hope that the release of sterile insects in the wild and their mating with the wild population will result in infertile eggs [1].

The possibility of sterile male-only releases in some species has made the SIT application more efficient and cost effective in several ways. As the probability of mating between sterile males and wild females is increased, the damage of fruits due to the stinging by sterile females is avoided and, moreover, the overall costs associated with releasing and monitoring are drastically reduced [2, 3]. Male-only releases have been possible due to the development of genetic sexing strains (GSS) [4]. The principal requirements for the construction of a GSS include a selectable marker (morphological and/or conditional lethal) and the pseudo-linkage of the wild type (rescue) allele of this marker (from an autosome carrying the wild allele) with the male determining region, which in tephritid species is located on the Y chromosome. After the application of an appropriated scheme of crosses and backcrosses, it is possible to identify individuals that have the dominant wild type allele pseudo-linked to the Y-chromosome, yielding a strain that produces males with the wild type phenotype and mutant females [4–8].

During the last 60 years, significant progress has been achieved for the development and application of SIT against diverse insect fruit fly pests, with the Mediterranean fruit fly *Ceratitis capitata* being the model species [9]. However, despite numerous studies on all aspects of the biology and ecology of *Anastrepha fraterculus* including mass rearing [10], quality control [11], gamma irradiation [12], mating compatibility among different populations [13, 14], pheromones, hybridization, cytology [15], genetics [16] and cytogenetics [17], in part because of the lack of appropriate strains, it has not yet been possible to use the SIT against this pest.

The Anastrepha genus is endemic to America and is the most diverse genus of the Tephritidae [18]. *A. fraterculus* (Wiedemann), commonly known as the South American fruit fly, is a species of major economic and quarantine importance. It attacks more than 80 host species [19] causing severe economic losses which may reach to 100% losses if control measures are not applied. Desirable control measures include the use of integrated pest management (IPM) programs incorporating environment-friendly techniques such as the SIT [20–22].

The lack of genetic sexing strains which would enable sterile male-only releases has prevented the development and large-scale implementation of SIT applications, similar to the ones of *Ceratitis capitata* and *Anastrepha ludens*, for control of *A. fraterculus*. In the present study, we present the isolation of three morphological mutations, one of which (black pupae) was used as a selectable for the construction and evaluation of the first genetic strains of *A. fraterculus sp. 1*.

## Results

### Morphological description and genetic analysis of mutants

**Fig. 1:**
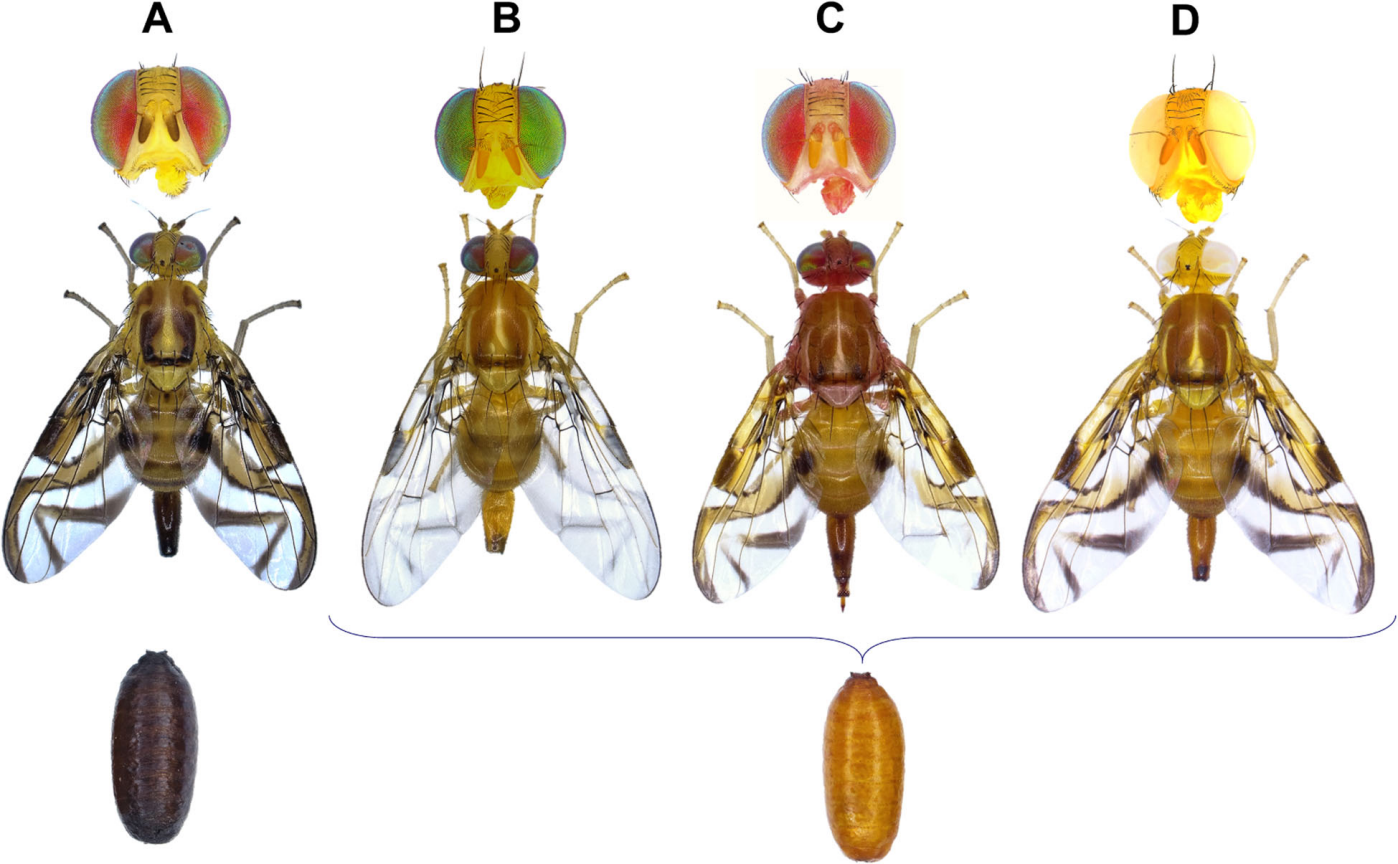
Phenotype of wild type and mutant individuals of *Anastrepha fraterculus sp. 1*

During a regular screening of a laboratory strain of *A. fraterculus sp. 1* (South of Brazil and Argentina), three mutations were discovered: *black pupae* (*bp*), *red body* (*rb*) and *white eye* (*we*). The black pupae phenotype was characterized by the black color of the pupae as well as the very dark color and wing veins at the adult stage compared to the wild type phenotype (Fig. 1a and b). The morphology of the *bp* mutants of *A. fraterculus sp. 1* was very similar to that described in the closely related species of *A. ludens* [8]. The red body phenotype is evident by the abnormal red body coloration only at the adult stage. At this stage, the phenotype was particularly pronounced in the light parts of the adult body and could easily be observed with naked eye (Fig. 1c). The white eye phenotype was characterized by the white colour of the adult eye and it was similar to that previously described in other species including *C. capitata* [23] and *A. ludens* [24] (Fig. 1d). Given that only the black pupae phenotype was expressed in an early developmental stage (pupal), the *bp* locus was chosen as a selectable marker for the development of a pupal color-based genetic sexing strain in *A. fraterculus sp. 1*.

Genetic analysis indicated that the inheritance of each of the mutant phenotypes is controlled individually by single autosomal, recessive genes (Table 1). Linkage analysis showed that only the cross between *rb* and *we* was consistent with the ratio expected in F_2_ for independently assorting genes (9 WT:3 *rb*:3 *we*:1 *rb we*). The crosses between *bp* to *rb* and *bp* to *we* resulted in slight deviations from the expected 9:3:3:1 ratio (Table 1). Backcrossing males from the GSS-89 strain (see below) with double mutant females carrying *rb* and *we* confirmed that these two loci are not linked to the *bp* locus (Table 1).

**Table 1:** (a) results of inheritance mode experiments of mutants, (b) linkage analysis of *red body* (*rb*), *white eye* (*we*) and *black pupae* (*bp*) mutants and (c) GSS backcrossing to *we* and *rb* alleles in *Anastrepha fraterculus sp. 1*

Inheritance crosses		F_1_ phenotype		F_2_ phenotypes				Total		X^2^ (3:1)
				Wild type		mutant				
*♂*	*♀*			*♂*	*♀*	*♂*	*♀*			
*rb*	WT	all population WT		302	297	91	88	778		1.65
WT	*rb*			240	188	76	87	591		2.10
*we*	WT			259	245	69	72	645		3.39
WT	*we*			119	108	33	31	291		1.40
*bp*	WT			528	511	190	194	1423		0.16
WT	*bp*			446	439	155	148	1188		2.99
Linkage crosses		F_1_ phenotype		F_2_ phenotypes				Total		X^2^ (9:3:3:1)
*♂*	*♀*			WT	mutant	mutant	double mutant			
*we*	*bp*	all population WT		997	320^1^	361^2^	75	1753		14.43
*rb*	*bp*			1002	344^3^	363^2^	82	1791		10.43
*rb*	*we*			559	192^3^	177^1^	61	989		0.63
Backcrossing		WT		*rb*		*we*		*rb we*		Total
*♂ F*_*1*_	*♀*	*♂*	*♀*	*♂*	*♀*	*♂*	*♀*	*♂*	*♀*	
GSS-89	*rb we*	250	241	181	183	231	211	216	187	1700

Hypothesis 3:1, X^2^_0.05, 1_ = 3.841

Hypothesis 9:3:3:1, X^2^_0.05, df = 3_ = 7.82

*WT* Wild type; mutant^1^ = *we;* mutant^2^ = *bp;* mutant^3^ = *rb*

### Development and characterization of pupal color-based genetic sexing strain (GSS)

Six hundred males were screened for the presence of irradiation-induced translocations which could result in a genetic sexing strain characterized by only males emerging from wild type brown pupae (T(Y;*bp*^*+*^)/*bp*) and only females from mutant black pupae (*bp/bp*). Four such males were identified, and these were used for the establishment of genetic sexing strains designated respectively as GSS-172, GSS-119, GSS-89 and GSS-33. The pupal color phenotype in relation to the sex was closely monitored in all four GSS, for eight generations. All recombinants (males emerged from black pupae and females emerged from brown pupae) representing translocation breakdown events were removed. The strains showed different recombination rates with the lowest one observed in GSS-89 (GSS-172 = 0.39%, GSS-119 = 0.71%, GSS-89 = 0.26%, GSS-33 = 0.72%). The recombination rate was consistently lower in males compared to females in all strains (Table 2).

**Table 2:** Percentage of recombination per generation of the different *Anastrepha fraterculus sp. 1* T(Y;*bp*^*+*^)/*bp* genetic sexing strains (GSS)

GSS	Generation	Male			Female			Total recombination (%)
		WT	*bp*	recombinant (%)	WT	*bp*	recombinant (%)	
172	Parentales	21	0	0.00	0	10	0.00	0.00
	F_1_	397	0	0.00	1	354	0.28	0.13
	F_2_	455	1	0.22	2	447	0.45	0.33
	F_3_	302	0	0.00	0	261	0.00	0.00
	F_4_	151	0	0.00	1	137	0.72	0.35
	F_5_	76	0	0.00	1	92	1.08	0.59
	F_6_	303	0	0.00	2	125	1.57	0.47
	F_7_	251	0	0.00	9	309	2.83	1.58
	F_8_	597	0	0.00	1	510	0.20	0.09
119	Parentales	15	0	0.00	0	14	0.00	0.00
	F_1_	87	0	0.00	1	70	1.41	0.63
	F_2_	254	0	0.00	1	276	0.36	0.19
	F_3_	245	0	0.00	2	237	0.84	0.41
	F_4_	218	0	0.00	0	169	0.00	0.00
	F_5_	83	1	1.19	2	36	5.26	2.46
	F_6_	271	0	0.00	3	103	2.83	0.80
	F_7_	634	0	0.00	22	718	2.97	1.60
	F_8_	870	0	0.00	6	887	0.67	0.34
89	Parentales	22	0	0.00	0	17	0.00	0.00
	F_1_	82	0	0.00	1	63	1.56	0.68
	F_2_	116	0	0.00	2	89	2.20	0.97
	F_3_	265	0	0.00	1	214	0.47	0.21
	F_4_	139	0	0.00	1	110	0.90	0.40
	F_5_	66	0	0.00	0	32	0.00	0.00
	F_6_	23	0	0.00	0	13	0.00	0.00
	F_7_	140	0	0.00	0	164	0.00	0.00
	F_8_	461	0	0.00	1	398	0.25	0.12
33	Parentales	8	0	0.00	0	5	0.00	0.00
	F_1_	18	0	0.00	0	9	0.00	0.00
	F_2_	44	0	0.00	0	28	0.00	0.00
	F_3_	72	2	2.70	2	69	2.82	2.76
	F_4_	29	0	0.00	0	14	0.00	0.00
	F_5_	79	1	1.25	2	51	3.77	2.26
	F_6_	409	0	0.00	4	203	1.93	0.65
	F_7_	437	0	0.00	3	331	0.90	0.39
	F_8_	263	0	0.00	2	178	1.11	0.45

**Fig. 2:**
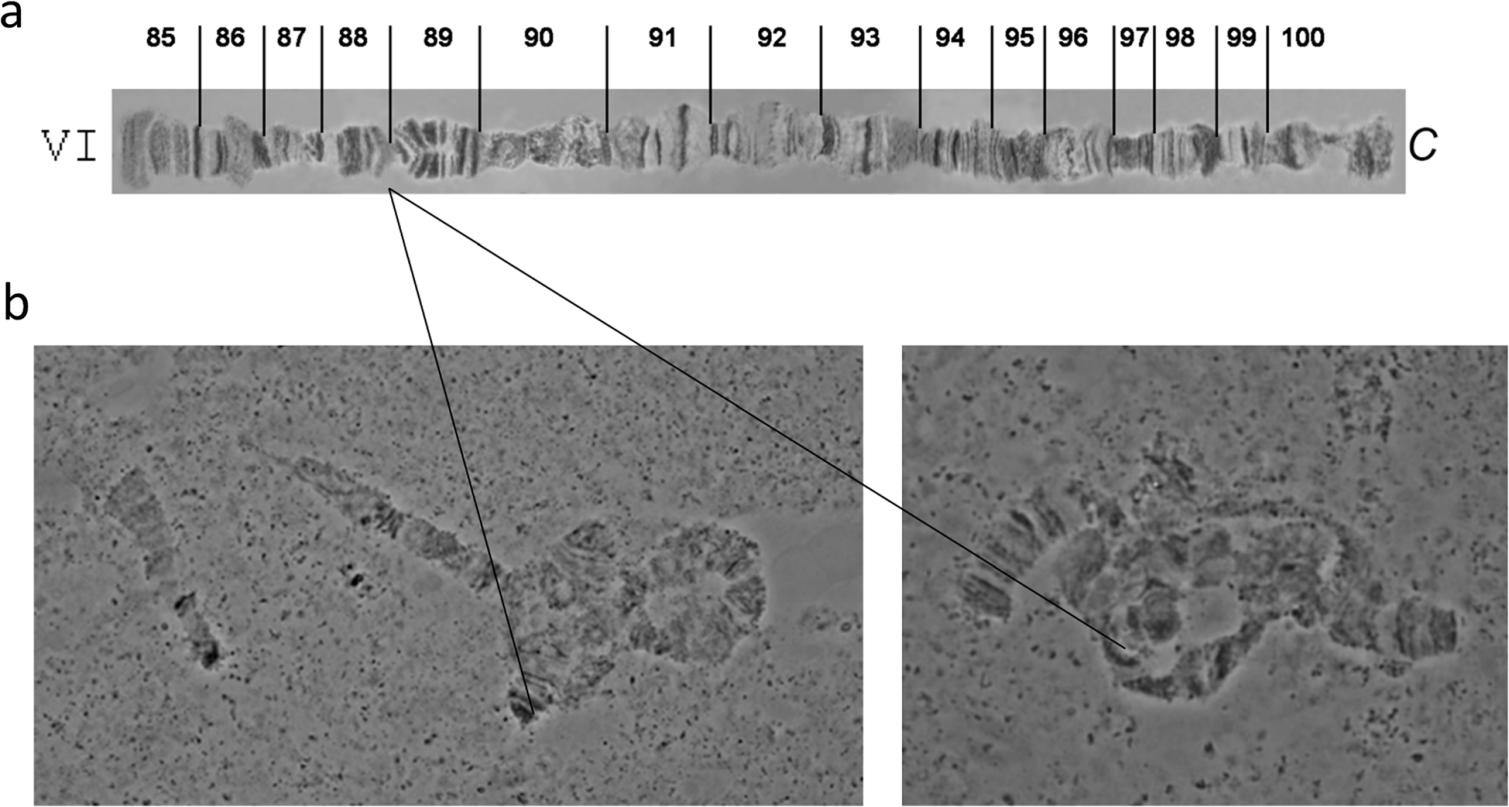
Polytene chromosome of the *Anastrepha fraterculus sp. 1* strain T[(Y;VI *bp*^+^)/*bp*]-89 (GSS-89) . **a** Reference map of chromosome VI (section 85–100). **b** The part of the VI chromosome which is involved in the (Y;A) translocation

Cytogenetic analysis of the GSS-89 confirmed previous studies that the autosomes II to VI are polytenized in the salivary glands while chromosomes X and Y do not polytenize due to their heterochromatic nature [16, 17]. The analysis also indicated that the (Y;A) translocation involves the smallest autosome and that the translocation breakpoint is located in band 88 according to the published map of polytene chromosomes of this species (Fig. 2).

### Biological characteristics

Comparative analysis between the wild type, *bp* and the four T(Y;*bp*^*+*^)/*bp* GSS strains revealed significant differences in respect to fertility (F_5,24_ = 12.86; *p* < 0.001), egg to pupa survival (F_5,24_ = 9.73; *P* < 0.001), pupae to adult survival (F_5,24_ = 3.23; *P* = 0.022) and overall fitness (F_5,24_ = 17.00; *P* < 0.01) (Table 3). For these biological characteristics, the wild type strain exhibited the best values followed by the *bp* mutant strain. The four GSS were inferior to the wild type and the *bp* strains in all parameters studied; however, of these, the strain designated as GSS-89 exhibited the best values with respect to fertility and overall fitness.

**Table 3:** Quality control indices (Mean ± SE) of different *Anastrepha fraterculus sp. 1* strains under laboratory rearing environment

Strain	Fertility (%)	Egg to pupae survival (%)	Pupa to adult survival (%)	Overall fitness
WT	81.00 ± 1.81 a	73.00 ± 1.34 a	96.72 ± 0.92 a	0.57 ± 0.01 a
*bp*	68.60 ± 2.03 b	49.00 ± 2.21 b	84.07 ± 5.01 ab	0.28 ± 0.01 b
GSS-172	45.50 ± 5.75 cd	29.00 ± 6.14 bc	86.84 ± 4.55 ab	0.12 ± 0.04 c
GSS-119	47.80 ± 3.93 cd	35.50 ± 5.94 bc	77.04 ± 4.04 b	0.14 ± 0.03 bc
GSS-89	54.20 ± 4.91 bc	29.80 ± 4.61 bc	83.30 ± 2.83 ab	0.14 ± 0.03 bc
GSS-33	37.20 ± 2.53 d	21.70 ± 2.35 c	90.72 ± 2.51 ab	0.07 ± 0.01 c

Overall fitness = (Fertility/100) (egg to pupae/100) (pupae to adult/100). For each column, lower case letters represent significant differences between strains (*P* < 0.05)

## Discussion

Three mutations were isolated in the present study to enrich the genetic tools available in this major agricultural pest species, the South American fruit fly *Anastrepha fraterculus sp.1*. Of the mutations recovered, the fact that the black pupae mutant phenotype is expressed at the pupal stage, much earlier that the red body and the white eye phenotypes expressed at the adult stage, was the key factor for its further characterization and selection as a selectable marker for the construction of the first genetic sexing strain in this species. Using this GSS, it becomes possible to remove females at the pupal stage during the mass rearing, and this in turn would allow SIT operational programmes to handle males-only during marking, packaging, irradiation, release and field monitoring. The use of GSS for male-only releases have been shown to improve the efficiency and cost-effectiveness of SIT in tephritid flies [9, 25, 26] and this approach is currently being used in action programs against two major pests the Mediterranean fruit fly, *Ceratitis capitata* and the Mexican fruit fly, *Anastrepha ludens*.

It is worth noting that a black pupae mutation of the type identified here was also used as a selectable marker for the development of a GSS (namely Tapachula-7) which is currently being used in SIT applications against *A. ludens* [8]. However, despite the fact that these are closely related species, the *bp* locus appears to be carried on different autosomes in each case. In *A. ludens* it is carried on chromosome 2 while in *A. fraterculus sp. 1,* it is found on chromosome VI in [27]. It may be that the black pupae phenotype has been induced in two different loci residing on different chromosomes in these species, but alternatively, these mutations may originate from the same gene residing on chromosomes that have undergone extensive rearrangement in evolution of these two species. To resolve this, more work remains to be done to clarify the extent of homology between all of the chromosomes in these two species.

For any genetic sexing strain, especially during rearing, the stability of the translocation is an important property. All such translocations are subject to some degree of breakdown as reflected in recombination or loss of the artificial linkage relationship generated for the purposes of genetic sexing. In other studies, this has been shown to depend greatly on the structure of translocation, mainly the distance between the translocation breakpoint and the selectable marker [28]. Data presented in this study showed that during a period of eight generations, the recombination rate was less than 1% (detected as the presence of black pupa males and brown pupae females) for all of the GSS produced here, with the lowest rate (0.29%) observed in GSS-89. Notably, in these cases, brown pupae females were more abundant than black pupae males. It should also be noted that this low recombination rate was recorded under small scale rearing conditions. Any such breakdown may significantly increase during mass rearing conditions and result in the risk of compromising the genetic stability of the GSS. However, the application of a filter rearing system designed to remove any recombinants at the early stages of the mass rearing process, and/or the incorporation of chromosome inversions, have both been shown to help ensure the genetic integrity of any GSS [29].

However, because of the (Y;A) translocation, only 50% of the sperm produced by males of the GSS are genetically balanced, and for this reason the GSS are considered as semi-sterile [4, 8, 30, 31]. The evaluation of the strains used in the present study showed that among all GSS developed here, the GSS-89 is the most fertile (about 54% fertility). This result, in combination with its low recombination rate, suggests that it could be a productive and genetically stable GSS under mass rearing conditions. However, it is strongly recommended that any GSS which will be used for mass rearing and male-only releases in an SIT operational programme should always be selected from a large number of translocation lines, each of which has been assessed with respect to their genetic stability and productivity.

It is also worth noting that, given the fact that *A. fraterculus* is a species complex consisting of at least eight morphotypes, it may be possible to develop and implement an appropriate genetic introgression scheme to transfer the mutant bp allele from one morphotype to another while at the same time largely maintaining their genetic integrity. A similar approach has been recently applied among some Bactrocera species [32]. Such an approach should significantly facilitate the development of pupal color based genetic sexing strains for each of the members of the *A. fraterculus* species complex.

## Conclusions

The present study reports on three novel morphological mutations in *A. fraterculus sp. 1*. One of these, the black pupae mutation, was used a selectable marker for the construction of the first genetic sexing strains in this species. Initially, four genetic sexing strains [T(Y;*bp*^*+*^)/*bp*] were developed and evaluated in respect to their genetic stability and productivity. From this, the strain designated as GSS-89 was chosen as being the most genetically stable and productive. As the selection is based on the pupal color, using this strain a robust sex separation system can also be established by using a color sorting machine. This would allow for male-only releases and would greatly facilitate the development and implementation of large scale operational SIT programmes against this important pest in South America.

## Methods

### Insects

During a routine screening, three new morphological markers (mutants) were isolated by J. S. Meza (JSM) and D. F. Segura (DFS) from *A. fraterculus sp. 1* population at the Insect Pest Control Laboratory (IPCL), Joint FAO/IAEA Division of Nuclear Techniques in Food and Agriculture, Seibersdorf, Vienna, Austria [33], and respective colonies of each of the mutant lines were established. These spontaneous mutations were designated as; *black pupae* – *bp* (JSM)*, red body – rb* (JSM) and *white eye – we* (DFS). All wild type and mutant colonies were maintained under an artificial rearing system as described by [10].

### Genetic analysis of the morphological mutations

Single pair matings between flies from the three mutant lines and wild type (WT) flies were performed reciprocally in order to determine the inheritance pattern. The F_1_ generation progeny were interbred in groups of five pairs to obtain the F_2_ generation, and the F_2_ phenotypes were recorded. In a separate experiment, crosses between mutants were carried out. The F_1_ generation progeny were interbred and the F_2_ phenotypes were recorded to assess their potential linkage relationships. In addition, double-homozygous mutant females (*rb we*) were back-crossed to T(Y;*bp*^*+*^)/*bp* males to assess the linkage relationships of *bp* to the *rb* and *we* loci.

### Generation of translocations for development of a pupal color-based genetic sexing strain (GSS)

One day before eclosion, pupae from the WT strain were gamma-irradiated at 30 Gy by using Gamma Cell Cobalt^60^. Irradiated WT males were mated with black pupae (*bp/bp*) females. Over 600 WT F_1_ males were individually backcrossed to five *bp/bp* females in small containers (families). The F_2_ phenotypes of each family were recorded and families potentially carrying translocation T(Y;*bp*^*+*^)/*bp* were identified as those having males emerged from brown pupae (WT) and females from black pupae [6, 8, 34]. Such families were used to develop the GSS by crossing, in each generation, brown pupae males to black pupae females and removing all recombinants (black pupae males and brown pupae females).

### Biological characteristics

The biological characteristics of the WT, *bp* mutant, and the genetic sexing strains (GSS-172, GSS-119, GSS-89 and GSS-33) were assessed by rearing the strains at 25 ± 1 °C. The collected eggs were incubated for 2 days in aerated water. After the incubation period, one thousand eggs from each strain were transferred on an artificial diet in groups of 200 eggs aligned on a small piece of cloth mesh. Three days after the transfer of the eggs to the diet, the number of eggs hatched were recorded to estimate the fertility. Ten days after the transfer of the eggs, the larvae were removed from the artificial diet and placed into a recipient tray with sawdust to complete the pupation (12 days) and during the separation of pupae from the sawdust by sieving, the number of mature pupae were placed in a Petri dish and recorded to estimate egg-to-pupa survival. The number of emerged adults was then recorded to estimate the pupa-to-adult survival.

### Cytogenetic analysis

Third instar male larvae were used for preparation of the salivary gland polytene chromosomes for analysis of the GSS-89 genetic sexing strain of *A. fraterculus sp. 1*, using the method described previously for *C. capitata* [35] and for *A. ludens* [36]. Briefly, the male larvae (identified based on the brown coloration of the anal lobes) were dissected in 45% acetic acid and transferred to 3 mol/L HCl for 1 min. Chromosomes were fixed in glacial acetic acid – water – lactic acid (3:2:1, respectively) for about 5 min before being stained in lactoacetic orcein for 10–15 min. Excess stain was removed by washing the glands in lacto-acetic acid before squashing. Chromosome slides were analyzed at 60x and 100x objectives in a phase contrast microscope (LEIKA DMR). Well spread nuclei or isolated chromosomes were photographed using a digital camera (ProgResCFcool JENOPTIC/JENA/Germany) [17].

### Data analysis

The genetic crosses data were evaluated using contingency tables and Pearson Chi-squared tests. Each biological characteristic was analyzed by one-way analysis of variance (ANOVA) using the “strain” as predictor of fertility, egg to pupa survival, pupa to adult survival and overall fitness [(Fertility/100)(egg-to-pupae/100)(pupae-to-adult/100)]. The Tukey’s HSD test was used as a post-hoc method to compare means between strains on significant factors. In order to normalize the data distribution and stabilize the variances, the data in percentages were transformed following $arcsine\sqrt{x+1}$ [37]. All data were analyzed with Statistical Discovery JMP 11.0.0 software (SAS institute).

## Data Availability

All data generated or analysed during this study are included in this published article.

## Abbreviations

SENASICA: Servicio Nacional de Sanidad, Inocuidad y Calidad
IICA: Instituto Interamericano de Cooperación para la Agricultura
FAO: Food and Agriculture Organization
IAEA: International Atomic Energy Agency
AW-IPM: Area-wide Integrated
SIT: Sterile Insect Technique
GSS: Genetic Sexing Strain
IPM: Integrated Pest Management
IPCL: Insect Pest Control Laboratory
WT: Wild Type
